# Data on fluoride concentration in drinking water resources in Iran: A case study of Fars province; Larestan region

**DOI:** 10.1016/j.dib.2018.05.112

**Published:** 2018-05-24

**Authors:** Mohammad Hadi Dehghani, Gholam Ali Haghighat, Mahmood Yousefi

**Affiliations:** a Department of Environmental Health Engineering, School of Public Health, Tehran University of Medical Sciences, Tehran, Iran; b Institute for Environmental research, Center for Solid Waste Research, Tehran University of Medical Sciences, Tehran, Iran

**Keywords:** Fluoride, Drinking water, Fars province, Larestan, Iran

## Abstract

Fluoride is a natural element among minerals, geochemical sediments and natural water systems which is entered to body chain by drinking water. Groundwater is the main and the best source of drinking water in southern areas of Iran especially in the cities of Lar and Gerash (Fars province). So due to the health significance fluoride including dental and skeletal fluorosis, fertility, abortion and thyroid diseases, etc., measuring has high importance in the water resources of this region of Iran. Fluoride concentration was 0.35–3.46 mg/L and 78.26% drinking water sources contains fluoride concentration above the WHO guideline.

**Specifications Table**

**Table:** 

Subject area	Water chemistry
More specific subject area	Water fluoride
Type of data	Tables, Figure
How data was acquired	Samples were examined in Water and Wastewater Laboratory of the Health Center of Shiraz Valfajr Shohadaye and had been assessed by sodium 2-(parasulfophenylazo)-1, 8-dihydroxy-3, 6-naphthalene disulfonate (SPADNS) colorimetric method based on Standard Methods. SPADNS standard methods with a spectrophotometer DR model 5000 of HACH Company
Data format	Raw, Analyzed
Experimental factors	Fluoride concentration above, in abstract section, were analyzed according to the standards for water and wastewater treatment handbook.
Experimental features	Determine the concentration levels of fluoride
Data source location	Larestan, Fars province, Iran
Data accessibility	The data are available whit this article

**Value of the data**

• Knowledge of fluoride level in potable groundwater is important for health care personnel and policymakers.
• Long-term consumption of drinking water with a high fluoride concentration leads to many adverse effects on human including dental and skeletal fluorosis [1], [2], [3], [4].
• Based on the data article, DE fluoridation of drinking water resource could be recommended in this region with high fluoride concentrations.

## 1 Data

Fluoride concentration (mg/L) in drinking water of Larestan region, fluoride concentration in drinking water according to Institute of Standards and Industrial Research of Iran are summarized in Table 1, Table 2 respectively.

**Table 1:** Fluoride concentration (mg/L) in drinking water of Larestan region, Fars province, Iran.

Number^a^	Fluoride concentration	Number^a^	Fluoride concentration	Number^a^	Fluoride concentration
	(mg/L)		(mg/L)		(mg/L)
1	2.38	32	0.67	63	1.07
2	1.92	33	0.62	64	1.3
3	2.64	34	0.35	65	1.41
4	2.42	35	0.63	66	0.91
5	2.14	36	0.64	67	0.76
6	1.54	37	0.65	68	1.14
7	1.56	38	1.65	69	1.28
8	2.66	39	0.8	70	1.96
9	2.84	40	0.92	71	2.35
10	2.66	41	0.85	72	2.29
11	2.86	42	1.33	73	1.83
12	3.46	43	0.45	74	2.02
13	3.44	44	1.48	75	1.63
14	2.36	45	1.53	76	1.08
15	3.28	46	1.13	77	1.72
16	2.08	47	1.1	78	2.48
17	2.28	48	1.28	79	2.73
18	2.95	49	1.39	80	1.84
19	2.8	50	1.7	81	1.65
20	2.3	51	1.9	82	2.1
21	2.58	52	1.59	83	1.95
22	1.62	53	0.77	84	2.1
23	2	54	0.51	85	2.88
24	2.4	55	0.86	86	1.23
25	0.8	56	1.65	87	1.52
26	0.64	57	0.83	88	1.44
27	0.96	58	0.85	89	1.67
28	0.64	59	2.02	90	1.46
29	0.96	60	1.71	91	1.43
30	2.02	61	1.43	92	2.73
31	0.71	62	1.7		
WHO guideline	0.5–1.5				

a Number of supply sources for drinking water.

**Table 2:** Fluoride concentration in drinking water, according to Institute of Standards and Industrial Research of Iran [5], [6], [7], [8], [9], [10].

The annual average maximum	The maximum allowable	Desirable level (mg/l)	The minimum allowable
daily air temperatures (°C)	amount of fluoride (mg/l)		amount of fluoride (mg/l)
10–12	2.4	1.2	1.1
12–14.6	2.2	1.1	1
14.6–17.7	2	1	0.9
17.7–21.5	1.8	0.9	0.8
21.5–26.3	1.6	0.8	0.7
26.3–32.5*	1.4*	0.7*	0.6*

* These target values accounts for Larestan region since the annual average maximum daily temperature is about 32.5 °C there.

## 2 Experimental design, materials and methods

### 2.1 Study area description

**Fig. 1:**
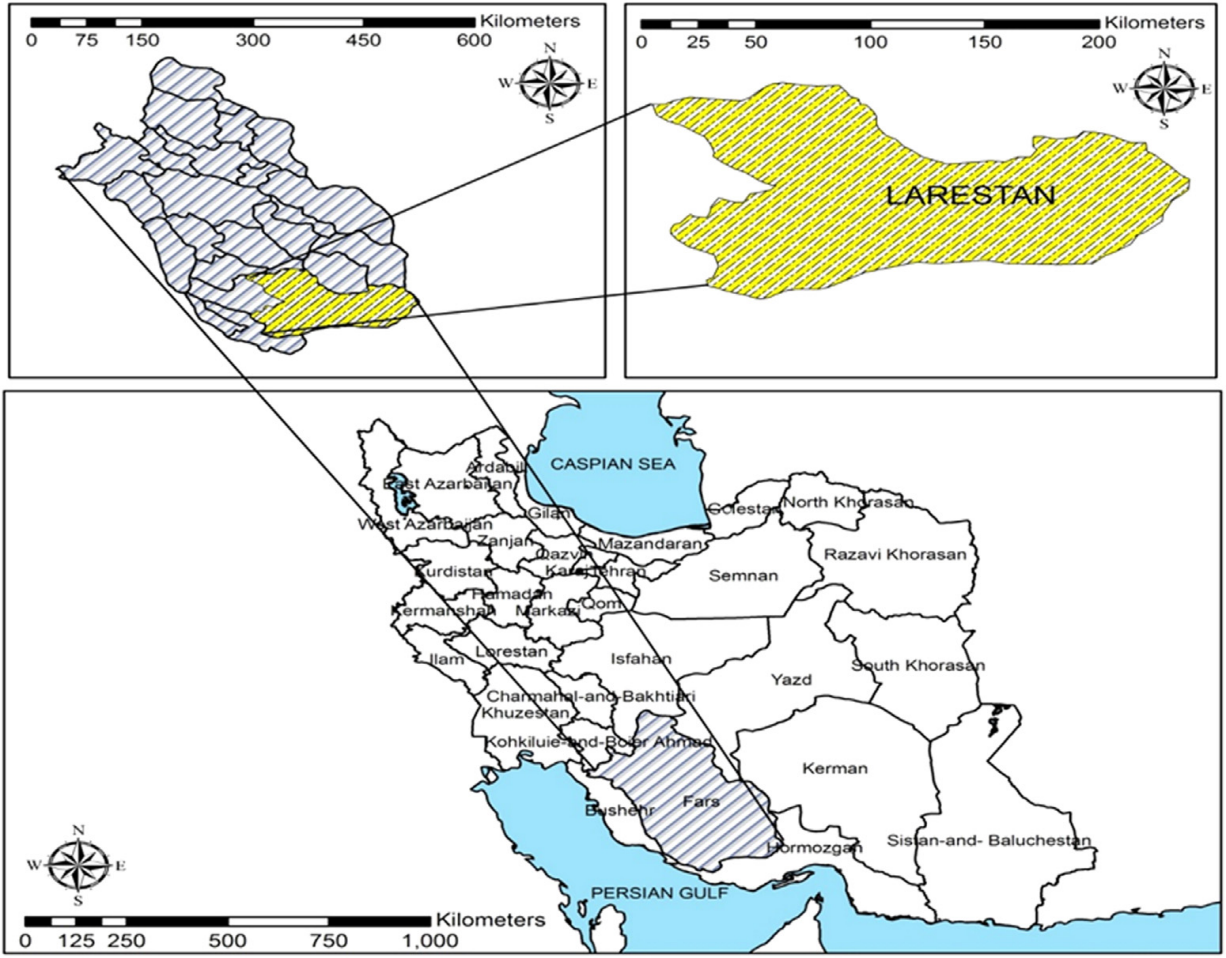
Location of the study area in Larestan city, Fars province, Iran.

Lar and Gerash are located between 27° and 60 min to 28° and 25 min׳ north latitude and 52° and 25 min to 55° and 38 min east of Greenwich meridian: the two cities were called Larestan city before, and are located in the southern province of Fars and were separated in 2011 during administrative divisions. It leads to Jahrom from north, to Darab and Zarin dasht from north-east, to Firouzabad from north-west, Ghirokarzine, Khange, and to the province of Hormozgan from south, west to the city of Lamerd and Mehr and east to the Finn of Bandar Abbas. The city with an average altitude of 900 m above sea level, is one of the hot and dry regions of Iran that is allocated 1.6 (17%) of Fars Province. Larestan has of hot and dry climate with mild winters and hot and very dry summers. Larestan rainfall was 198.8 mm during the 20-year statistics of Larestan airport weather station. The highest annual precipitation is related to January and February that precipitations were long but had low intensity. In July and August rainfalls were at minimum level with short time of raining and high intensity (Fig. 1).

### 2.2 Sample collection and analytical procedures

92 wells have been selected as randomly and were sampled regularly Four times a year from 2010 to 2011. Samples were examined in Water and Wastewater Laboratory of the Health Center of Shiraz Valfajr Shohadaye and had been assessed by sodium 2-(parasulfophenylazo)-1, 8-dihydroxy-3, 6–naphthalene disulfonate (SPADNS) colorimetric method based on Standard Methods. SPADNS standard methods with a spectrophotometer DR model 5000 of HACH Company [11], [12], [13], [14], [15], [16]. Analytical method for fluoride determination between the 0.0625–1.75 mg L^−1^ (*r*=0.9993) and the higher level of this range were diluted and measured. The fluoride concentration was assessed by Spectrophotometer (DR/5000, USA) and obtained limits of determination (LOD) and quantification (LOQ) were 0.12 ppm and 0.37 ppm respectively [1], [17], [18], [19], [20], [21], [22], [23], [24]. This method is acceptable for USEPA and can be reported after the needed analyzes [1], [25], [26]. We used two 10 mm cubic cell in this method, one for the deionized water and the other for the sample. Then we added 2 mL SPADNS solution to each cell and keep stirring for a minute. Finally, we read each cell with the spectrophotometer.
